# Longitudinal study of inbreeding effect on growth traits of Marwari lambs

**DOI:** 10.5194/aab-68-541-2025

**Published:** 2025-09-01

**Authors:** Jayesh Vyas, Ashish Chopra, Harvinder Kumar Narula

**Affiliations:** 1 Animal Genetics and Breeding Division, ICAR-National Dairy Research Institute, Karnal 132001, Haryana, India; 2 Animal Genetics and Breeding Division, ICAR-Central Sheep and Wool Research Institute-Arid Region Campus, Bikaner 334001, Rajasthan, India; 3 Division of Animal Husbandry, Indian Council of Agricultural Research, New Delhi 110114, India

## Abstract

This study aimed to assess the impact of inbreeding on growth traits and genetic diversity in a closed flock of Marwari sheep. Data from 11 126 lambs born between 1981 and 2020 at ICAR-CSWRI-ARC in Bikaner, Rajasthan (India), were used to analyse trends in inbreeding, its impact on growth performance, and its effect on genetic diversity. The mean inbreeding coefficients for the entire population, males, and females were 1.55 %, 1.57 %, and 1.53 %, respectively. The overall proportion of inbred animals was 67.18 %, with the majority (38.21 %) exhibiting inbreeding levels below 5 %. The linear regression analysis revealed that, for every 1 % increase in the inbreeding coefficient, there was a corresponding change of 6, 79, 121, 146, 116 g, 0.78, and 0.38 g d^−1^ in birth weight (BWT), weight at 3 months (3 MWT), weight at 6 months (6 MWT), weight at 9 months (9 MWT), weight at 12 months (12 MWT), average daily gain from birth to weaning (ADG1), and from weaning to 6 months (ADG2), respectively. The regression analysis showed highly significant and positive effects on most growth traits except for KR3, GE3, and RGR3, with significant and negative effects. The ratio of the effective founder genome to ancestor ( $f_{g}/f_{a}$ ) revealed that 36 % of the original ancestral genetic diversity was retained in the reference population. A loss of 3 % in genetic diversity was attributed to the uneven contribution of founders, while bottleneck effects were due to the imbalanced contribution of breeding animals to the population's gene pool. Under a specified criterion, the rate of inbreeding gradually rose. The study revealed that, although a low level of inbreeding positively influenced all growth traits, it also impacted genetic diversity within the flock. To sustainably manage the population, utilizing a broader base of unrelated breeding rams and implementing scientifically guided mating strategies to improve performance while conserving genetic variability are recommended.

## 1 Introduction

Small ruminant rearing is the backbone of the rural economy, sustaining the rural people's livelihood and nutritional security, particularly in the harsh climatic conditions and rugged terrains of Rajasthan. India has various sheep and goat genetic resources adapted to its diverse agro-climatic regions. The farmers prefer small ruminant farming due to its specific advantages, mostly lower financial and labour investment, easy keep-up, availability of suitable local markets, faster return, etc., over other livestock species. The ever-increasing human population, faster urbanization, and increased purchasing capacity have led to a massive demand for meat, milk, wool, and other animal products in the country. The Marwari sheep is an important sheep breed in the hot, arid zone of Rajasthan as it produces medium- to coarse-quality carpet wool and mutton. It is the largest breed in the country's northwestern region, well recognized for its drought resistance capability, well adapted to harsh climates, and able to travel a greater range in search of forage (CSWRI breed profile, 2024).

Distribution of genetically superior breeding rams to farmers is a key strategy for improving the genetic quality of sheep flocks. To determine optimal breeding strategies to increase the efficiency of sheep farming, knowledge of inbreeding is essential to evaluate the growth performance under the breeding programme and to keep the inbreeding under acceptable limits. Inbreeding is a valuable strategy for genetic improvement by increasing the abundance of beneficial genes. Due to selection and the closed structure of flocks, intensive use of limited sires and dams can increase inbreeding and decrease genetic diversity among livestock populations (Boon, 2014). Inbreeding is desirable for selecting favourable alleles in the population, but it can be harmful if it exceeds acceptable limits (1 % per generation) (FAO, 1998). Many researchers have observed decreased performance in traits related to productivity, reproduction, fitness, and health in various sheep breeds as a result of inbreeding (Vyas et al., 2022; Rahim et al., 2023; Saran et al., 2024). Inbreeding is generally challenging to avoid in a small, closed, and selected population. Monitoring inbreeding trends is widely used to assess the flock's genetic drift rate.

The inbreeding depression is the main consequence of inbreeding, which causes a lowering in phenotypic values of production traits in inbred animals. This phenomenon has been documented across all livestock species (reviewed by Leroy, 2014; Doekes et al., 2021). Despite specific beneficial inbreeding results in selection schemes, breeders are conscious of its detrimental impacts and work to prevent it (Nilson et al., 2023; Vyas et al., 2023). Estimating the inbreeding coefficient “ F ”, which is defined as the likelihood that two alleles at a locus are similar by descent, is one of the methods for calculating inbreeding (Wright, 1922) and its impact on the trait of interest. Inbreeding coefficients have been accurately estimated in populations where parents have been identified for decades. Mating and management systems highly effect some demographic parameters, resulting in a severe loss of genetic diversity due to inbreeding. Maintenance of genetic diversity is important, reflecting the physiological, behavioural, and morphological differences seen in populations and among individuals (Illa et al., 2020).

Pedigree information helps to maintain inbreeding at a minimal level and genetic variability in the population (Santana et al., 2016). Calculating the inbreeding coefficient for many populations is challenging as it typically necessitates detailed pedigree information. However, in populations where parentage is known over multiple generations, accurate estimates of inbreeding coefficients can be achieved. There is a lack of information on growth efficiency traits in indigenous breeds. Therefore, this study was designed to assess the level of inbreeding, the proportion of inbred animals, and the impact of inbreeding on growth in the closed flock of Marwari sheep over 4 decades.

## 2 Materials and methods

### 2.1 Ethical approval

The present study was conducted using phenotypic records maintained at the Arid Region Campus ICAR–Central Sheep and Wool Research Institute, Beechwal, Bikaner (Rajasthan), India. Hence, it did not require any ethical approval.

### 2.2 Data collection and management practices

Information utilized in the study was collected from the pedigree records of 11 126 lambs maintained at ICAR–Central Sheep and Wool Research Institute, Arid Region Campus (ICAR-CSWRI-ARC), Beechwal, Bikaner, during the period from 1981 to 2020 to compute the inbreeding coefficient of each animal and for the characterization of genetic diversity. The research project was started in August 1981 under the All India Coordinated Research Project (AICRP). This project was converted into a Network Project in 1990 as a breeding project. The Marwari sheep have been improved through selection since the start of the project. At present, there are 300 breedable ewes in the flock. Concentrate mixture was offered ad lib to suckling lambs from the 15th day of age till weaning (90 d). After about 3 weeks from birth, lambs were sent for grazing in the morning and evening. In addition to 8–10 h of grazing and dry-fodder supplementation, 300 g of concentrate mixture was provided to all throughout the post-weaning period.

### 2.3 Pedigree analysis

Pedigree analysis of 11 126 lamb records (1981–2020) was performed using ENDOG v4.8 (Gutiérrez and Goyache, 2005) to estimate the number of generations across birth years.

### 2.4 Ancestral inbreeding coefficients

The inbreeding coefficient ( F ) was calculated according to the method outlined by Meuwissen and Luo (1992), employing the Pedigree Viewer V6.5b computer package (Kinghorn, 1994). Different ancestral inbreeding coefficients were estimated to determine whether alleles became identical by descent (IBD) for the first time or if they were already homozygous in previous generations. Gene-dropping, a straightforward computer simulation method widely used for estimating inbreeding levels and assessing genetic variability within animal populations, was employed for these calculations. The GRain v2.2 program (Baumung et al., 2015) employs stochastic gene-dropping to calculate ancestral inbreeding metrics from large, complex pedigrees. The pedigree-purging-based inbreeding coefficients estimated include the ancestral inbreeding coefficient by Ballou ( $F_{\mathrm{BAL}}$ ) (Ballou, 1997), the coefficients established by Kalinowski et al. (2000) and revised by Doekes et al. (2020) ( $F_{\mathrm{KAL}}$ and $F_{\mathrm{NEW}}$ , where $F_{\mathrm{NEW}}=F-F_{\mathrm{KAL}}$ , with F representing Wright's inbreeding coefficient), and the ancestral history coefficient ( $A_{\mathrm{HC}}$ ) (Baumung et al., 2015). These coefficients were calculated using 1 000 000 replications of the gene-dropping approach in the GRain v2.2 program.

The $F_{\mathrm{BAL}}$ coefficient represents the likelihood that an allele in an individual has been homozygous in any previous generation. The $F_{\mathrm{KAL}}$ identifies the portion of the genome where alleles are currently in IBD state and were also IBD in at least one ancestor. However, $F_{\mathrm{NEW}}$ estimates the share of alleles that become identical by descent for the first time. The ancestral history coefficient ( $A_{\mathrm{HC}}$ ) indicates the frequency with which a randomly chosen allele has reached identical-by-descent status during gene-dropping simulations in the pedigree.

Pearson's correlation coefficients and first-order partial correlation coefficients, combined with an information-theory-based approach (Reverter and Chan, 2008), were employed to detect significant associations among the various inbreeding coefficients. The calculation of first-order partial correlations and the application of information theory were carried out using the “PCIT” package (Watson-Haigh et al., 2010) in a statistical computing environment (R core team, 2024; version 4.4.3). After identifying Pearson correlations and significant associations, the inbreeding coefficient networks were analysed and visualized using Cytoscape 3.10.3 (Shannon et al., 2003).

### 2.5 Regression analysis

Data were categorized based on the inbreeding coefficient into the following classes: F=0 (non-inbred), 0>F<1.25 , 1.25>F<5 , and F>5 . Linear regression analyses of all growth traits concerning inbreeding were performed using IBM SPSS version 25.0 (IBM Corp., 2019). Inbreeding depression was estimated by regressing body weight traits on individual inbreeding coefficients, with generation included as a fixed effect in the model:

$$y_{ijk}=\mu +\beta F_{i}+{\mathrm{GEN}}_{j}+e_{ijk},$$

where $y_{ijk}$ is the observed growth trait of the k th lamb with i th inbreeding in the j th generation, μ is the overall population mean, $F_{i}$ is the inbreeding coefficient of the individual, GEN_*j*_ is the fixed effect of the j th generation, β is the regression coefficient representing the effect of inbreeding, and $e_{ijk}$ is the residual error (NID 0, $\sigma_{e}^{2}$ ). The data were divided into 10 periods, each consisting of 4 years (P1 (1981–1984), P2 (1985–1988), P3 (1989–1992), P4 (1993–1996), P5 (1997–2000), P6 (2001–2004), P7 (2005–2008), P8 (2009–2012), P9 (2013–2016), and P10 (2017–2020)).

The different economic growth traits used for the analysis were birth weight (BWT), 3-month or weaning weight (3 MWT), 6-month weight (6 MWT), 9-month weight (9 MWT), and 12-month weight (12 MWT). Average daily gain (in g d^−1^) from birth to weaning (ADG1), from weaning to 6 months (ADG2), and from 6 to 12 months (ADG3); Kleiber ratio from birth to 3 months or weaning (KR1), 3 months to 6 months (KR2), and 6 months to 12 months (KR3); growth efficiency from birth to weaning (GE1), from weaning to 6 months (GE2), and from 6 to 12 months (GE3); relative growth rate from birth to weaning (RGR1), from weaning to 6 months (RGR2), and from 6 to 12 months (RGR3) were calculated.

Average daily gain was calculated as follows: ADG1 = ((3 MWT - BWT) / 90) × 1000, ADG2 = ((6 MWT - 3 MWT) / 90) × 1000, and ADG3 = ((12 MWT - 6 MWT) / 180) × 1000. The Kleiber ratio has been suggested as a useful measure for feed efficiency in situations with limited inputs, offering insight into the economic efficiency of animal growth (Vyas et al., 2023). The Kleiber ratio was computed as follows: KR1 = ADG1 / (3 MWT)^0.75^, KR2 = ADG2 / (6 MWT)^0.75^, and KR3 = ADG3 / (12 MWT)^0.75^ (Kleiber, 1947). Growth efficiency was calculated as follows: GE1 = ((3 MWT - BWT) / BWT) × 100, GE2 = ((6 MWT - 3 MWT) / 3 MWT) × 100, and GE3 = ((12 MWT12 - 6 MWT) / 6 MWT) × 100. Relative growth rate was computed as follows: RGR1 = (Log_e_ (3 MWT) - Log_e_ (BWT)) / 90) × 100, RGR2 = (Log_e_ (6 MWT) - Log_e_ (3 MWT)) / 90) × 100, and RGR3 = (Log_e_ (12 MWT) - Log_e_ (6 MWT)) / 180) × 100.

### 2.6 Genetic diversity (GD)

The extent of genetic variation in the pre-defined reference population compared to the base population was estimated using the formula proposed by Lacy (1989, 1995).

$$\mathrm{GD}=1-\frac{1}{2f_{g}}$$

Genetic diversity in the base population was calculated as

$${\mathrm{GD}}^{*}=1-\frac{1}{2f_{e}}.$$

The genetic diversity lost in the founder generation was estimated using 1 - GD. In contrast, the loss of genetic diversity due to the unequal distribution of the founder's alleles was estimated using 1 - GD^*^. The disparity between GD^*^ and GD was determined using the approach outlined by Caballero and Toro (2000). The effective number of founders ( $f_{e}$ ) represents the number of founders that would equally contribute to the genetic diversity seen in the current population. In contrast, the founder genome equivalent ( $f_{g}$ ) reflects the genetic diversity remaining after accounting for the loss caused by genetic drift in small populations, even if all ancestors contributed equally (Lacy, 1989). Lambs born between 2017 and 2020 were designated as the reference cohort for calculating the genetic diversity parameter.

## 3 Results

**Figure 1:**
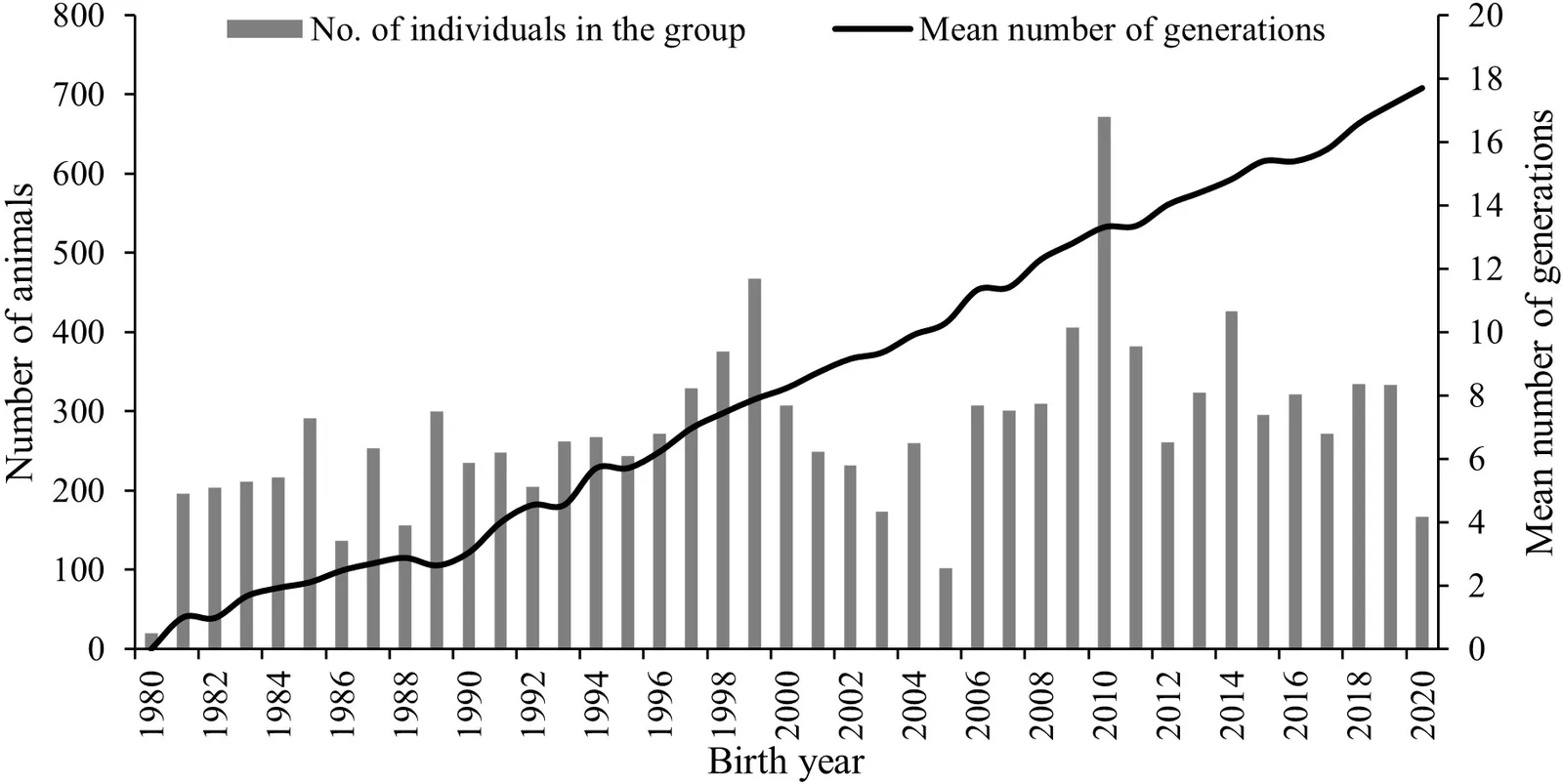
Trend in number of animals born and average number of generations per year in Marwari flock.

The Marwari sheep are primarily bred for carpet wool and mutton production and are maintained at a regional station in an arid and semi-arid environment. The nucleus structure and closed nature of the flock increase the risk of inbreeding across generations, which can reduce genetic diversity if not carefully managed. Pedigree analysis revealed trends in inbreeding and the extent of genetic diversity within the Marwari sheep population. The total number of animals born during the study period (11 126) and the mean number of generations (8.44) showed an increasing trend over the years analysed (Fig. 1).

**Figure 2:**
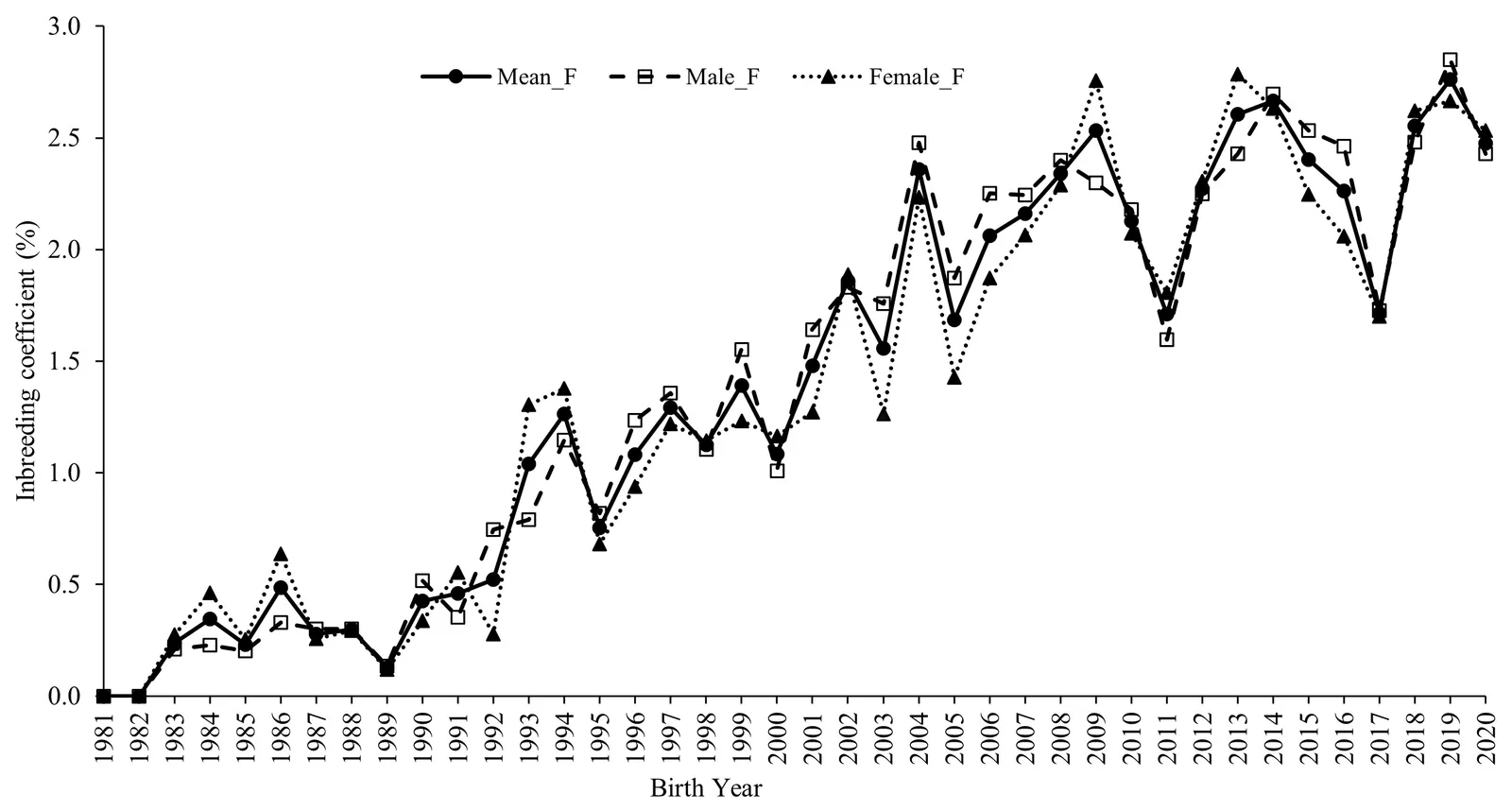
Trend in inbreeding over the years in the whole population and in male and female Marwari lambs.

The average inbreeding coefficient ( F ) for males and females across different periods (Table 1) indicates that the mean inbreeding coefficient ( F ) in the flock increased steadily over time, stabilizing at approximately 2.5 % over the last 7 years (Fig. 2).

**Table 1:** Inbreeding coefficient in whole population and in male and female Marwari lambs during different periods.

Period of birth	No. of animals			Mean inbreeding coefficient ( F %)		
	Total	Male	Female	Mean	Male	Female
P1 (1981–1984)	827	450	377	0.15	0.11	0.20
P2 (1985–1988)	731	352	379	0.30	0.28	0.32
P3 (1989–1992)	914	455	459	0.38	0.43	0.33
P4 (1993–1996)	1037	522	515	1.04	0.99	1.09
P5 (1997–2000)	1482	743	739	1.24	1.28	1.19
P6 (2001–2004)	931	497	434	1.84	1.94	1.73
P7 (2005–2008)	1020	515	505	2.14	2.25	2.03
P8 (2009–2012)	1720	874	846	2.15	2.10	2.20
P9 (2013–2016)	1358	701	657	2.50	2.54	2.45
P10 (2017–2020)	1107	578	529	2.40	2.38	2.41
Overall	11126	5686	5440	1.55	1.57	1.53

The average inbreeding coefficients in the whole population and in males and females were 1.55 %, 1.57 %, and 1.53 %, respectively. During the first period (1981–1984), the mean inbreeding coefficients for the whole population, males, and females were 0.11 %, 0.15 %, and 0.20 %, respectively. By the last period (2017–2020), these values had increased to 2.40 %, 2.38 %, and 2.41 %, respectively.

**Figure 3:**
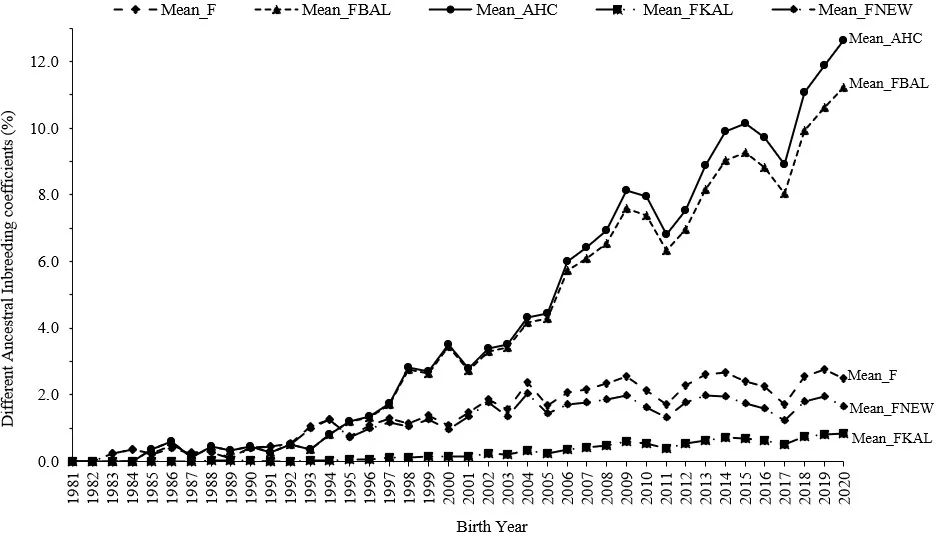
The evolution of different ancestral inbreeding coefficients in Marwari flock over the years.

Calculating ancestral inbreeding coefficients helps determine whether alleles became identical by descent for the first time or were already homozygous in earlier generations. In addition to the traditional inbreeding coefficient, various ancestral inbreeding coefficients were measured to determine whether inbreeding occurred recently or in the past. Among lambs with phenotypic data, the mean inbreeding coefficients for F , $F_{\mathrm{BAL}}$ , $A_{\mathrm{HC}}$ , $F_{\mathrm{KAL}}$ , and $F_{\mathrm{NEW}}$ were observed to be 1.55 %, 4.09 %, 4.40 %, 0.28 %, and 1.14 %, respectively (Fig. 3).

Pearson's correlations between all inbreeding coefficients were calculated (Table 2), and all correlation values were found to be highly significant ( p<0.01 ). Strong correlations were noticed between F and $F_{\mathrm{KAL}}$ and between F and $F_{\mathrm{NEW}}$ , as well as between $F_{\mathrm{BAL}}$ and $A_{\mathrm{HC}}$ and between $F_{\mathrm{KAL}}$ and $F_{\mathrm{NEW}}$ . Moderate correlations were found between F and $F_{\mathrm{BAL}}$ , F and $A_{\mathrm{HC}}$ , and $F_{\mathrm{BAL}}$ and $F_{\mathrm{NEW}}$ . In contrast, a weak correlation was identified between $A_{\mathrm{HC}}$ and $F_{\mathrm{NEW}}$ . Varying degrees of association among the coefficients suggest that each captures different aspects of inbreeding within the population.

**Table 2:** Pearson's correlation coefficients between different ancestral inbreeding coefficients.

Parameters	F	$F_{\mathrm{BAL}}$	$A_{\mathrm{HC}}$	$F_{\mathrm{KAL}}$	$F_{\mathrm{NEW}}$
F	1				
$F_{\mathrm{BAL}}$	0.40^**^	1			
$A_{\mathrm{HC}}$	0.40^**^	0.99^**^	1		
$F_{\mathrm{KAL}}$	0.72^**^	0.72^**^	0.71^**^	1	
$F_{\mathrm{NEW}}$	0.98^**^	0.30^**^	0.29^**^	0.60^**^	1

^**^ Highly significant. F denotes pedigree-based inbreeding coefficient from all of the genealogy, $F_{\mathrm{BAL}}$ denotes pedigree-based inbreeding coefficient (Ballou, 1997), $F_{\mathrm{KAL}}$ denotes pedigree-based inbreeding coefficient (Kalinowski et al., 2000), $F_{\mathrm{NEW}}$ denotes recent pedigree-based inbreeding coefficient calculated from gene drop (Doekes et al., 2019), and $A_{\mathrm{HC}}$ denotes ancestral history coefficient.

The inbreeding coefficient ( F ) and the proportion of inbred animals exhibited a linear increase over the observed periods. The population's overall proportion of inbred animals was 67.18 % (Table 3). Inbred animals during the first period (1981–1984) reached 1.21 %, which increased significantly to 98.33 % during the seventh period (2005–2008). In the present study, the overall proportion of outbred animals was 32.82 %, which was higher in initial periods but declined in subsequent periods due to scientific mating plans and management practices. In the most recent period, 80 % of the animals exhibited some degree of inbreeding. Among the total inbred animals, 23.83 % had inbreeding levels up to 1.25 %, and 38.21 % had levels up to 5 %, whereas only 4.96 % had more than a 5 % level of inbreeding. During the sixth period (2001–2004), 7.41 % of animals exhibited more than 5 % inbreeding, while 57.47 % had inbreeding levels up to 1.25 %.

**Table 3:** Percentage of inbred animals over the periods in Marwari flock.

Period of birth	Proportion of inbred animals (%)	Proportion among inbred animals ( F %)			
		F=0 (non-inbred)	0>F<1.25	1.25>F<5	F>5
P1 (1981–1984)	1.21 (10)	98.79	0	0	1.21
P2 (1985–1988)	4.51 (33)	95.48	0	2.33	2.19
P3 (1989–1992)	9.84 (90)	90.15	3.50	3.94	2.41
P4 (1993–1996)	40.50 (420)	59.49	23.53	10.51	6.46
P5 (1997–2000)	82.79 (1227)	17.21	58.43	18.76	5.60
P6 (2001–2004)	97.31 (906)	2.69	57.47	32.44	7.41
P7 (2005–2008)	98.33 (1003)	1.67	35.19	56.57	6.57
P8 (2009–2012)	92.09 (1584)	7.91	22.79	64.36	4.94
P9 (2013–2016)	94.18 (1279)	5.81	10.09	78.20	5.89
P10 (2017–2020)	82.38 (912)	17.54	7.50	68.44	6.51
Overall	67.18 (7464)	32.82	23.83	38.21	4.96

Number of observations is given in parentheses.

The regression analysis of growth traits with inbreeding is presented (Table 4). The overall estimates of birth (BWT), 3-month (3 MWT), 6-month (6 MWT), 9-month (9 MWT), and 12-month (12 MWT) weight were observed to be 3.03 ± 0.005, 14.59 ± 0.035, 20.49 ± 0.054, 23.72 ± 0.054, and 26.12 ± 0.054 kg, respectively. The overall estimates of average daily gain from birth to weaning (ADG1), weaning to 6 months (ADG2), and 6 months to 12 months (ADG3) were observed to be 128.52 ± 0.054, 66.05 ± 0.054, and 35.33 ± 0.054 g d^−1^, respectively. The Kleiber ratios from birth to weaning (KR1), weaning to 6 months (KR2), and 6 months to 12 months (KR3) were observed to be 16.95 ± 0.054, 6.66 ± 0.054, and 3.02 ± 0.054, respectively. The overall estimate of growth efficiencies from birth to weaning (GE1), weaning to 6 months (GE2), and 6 months to 12 months (GE3) were observed to be 385.13 ± 1.13, 41.49 ± 0.21, and 33.16 ± 0.20, respectively. The overall estimates of relative growth rate from birth to weaning (RGR1), weaning to 6 months (RGR2), and 6 months to 12 months (RGR3) were observed to be 1.73 ± 0.003, 0.38 ± 0.002, and 0.16 ± 0.001, respectively.

**Table 4:** Mean and regression coefficients of different growth traits in Marwari lambs.

Trait	Number	Mean ± SE	Regression with inbreeding coefficient				
			Constant	b	SE of b		
BWT (kg)	11111	3.03 ± 0.005	2.87	0.006^**^	0.002		
3 MWT (kg)	10279	14.59 ± 0.04	12.06	0.079^**^	0.015		
6 MWT (kg)	8922	20.49 ± 0.05	16.17	0.121^**^	0.023		
9 MWT (kg)	7132	23.72 ± 0.06	18.45	0.146^**^	0.028		
12 MWT (kg)	5928	26.12 ± 0.08	20.29	0.116^**^	0.032		
ADG1 (g d^−1^)	10239	128.52 ± 0.36	102.52	0.782^**^	0.158		
ADG2 (g d^−1^)	8690	66.05 ± 0.33	47.76	0.383^**^	0.150		
ADG3 (g d^−1^)	5578	35.33 ± 0.21	30.79	- 0.053^NS^	0.094		
KR1	10239	16.95 ± 0.02	15.59	0.037^**^	0.009		
KR2	8690	6.66 ± 0.03	5.84	0.008^NS^	0.012		
KR3	5578	3.02 ± 0.01	3.14	- 0.015^*^	0.007		
GE1	10239	385.13 ± 1.13	324.69	1.427^**^	0.504		
GE2	8690	41.49 ± 0.21	37.81	0.001^NS^	0.100		
GE3	5578	33.16 ± 0.20	36.87	- 0.277^**^	0.095		
RGR1	10239	1.73 ± 0.003	1.58	0.003^**^	0.001		
RGR2	8690	0.38 ± 0.002	0.34	0.001^NS^	0.001		
RGR3	5578	0.16 ± 0.001	0.17	- 0.001^**^	0.001		

^*^ Significant ( p≤0.05 ). ^**^ Highly significant ( p≤0.01 ). ^NS^ Non-significant. SE denotes standard error, BWT denotes birth weight, 3 MWT denotes weaning or 3-month weight, 6 MWT denotes 6-month weight, 9 MWT denotes 9-month weight, 12 MWT denotes 12-month weight, ADG1 denotes average daily gain from birth to 3 months, ADG2 denotes average daily gain from 3 to 6 months, ADG3 denotes average daily gain from 6 to 12 months, GE1 denotes growth efficiency from birth to 3 months, GE2 denotes growth efficiency from 3 to 6 months, GE3 denotes growth efficiency from 6 to 12 months, RGR1 denotes relative growth rate from birth to 3 months, RGR2 denotes relative growth rate from 3 to 6 months, and RGR3 denotes relative growth rate from 6 to 12 months.

The estimated values for founder genome equivalents ( $f_{g}$ ) and effective founders ( $f_{e}$ ) and ancestors ( $f_{a}$ ) were 16.76, 80, and 46, respectively, in the present population. The estimate for the $f_{g}/f_{a}$ ratio was 0.36. The estimate of GD in the reference population relative to the base population was 0.970. However, the genetic diversity estimated for the base population (GD^*^) was 0.993, and the estimate of GD^*^–GD differences was only 0.023. The losses of heterozygosity due to genetic drift and bottleneck effects (1 - GD) and uneven contributions of founder alleles (1 - GD^*^) were 0.03 and 0.007, respectively, in the founder generation.

## 4 Discussion

The objective of this study was to evaluate genetic diversity, inbreeding levels, trends, and their impact on growth traits in the Marwari flock based on pedigree and phenotypic records. Inbreeding estimates are primarily influenced by pedigree depth and completeness. Pedigree analysis serves as an effective tool for understanding the genetic structure of livestock populations and for identifying genetic erosion that affects genetic diversity (Addo et al., 2017; Vostra-Vydrova et al., 2020). However, the completeness of pedigree data significantly influences the accuracy of population parameter estimates, especially the level and trend of inbreeding (Justinski et al., 2023). Various methods are available for estimating inbreeding depression, with the standard approach being the regression of individual performance with pedigree-based inbreeding coefficients (Hossein-Zadeh, 2012). Our results obtained for pedigree completeness and inbreeding levels were consistent with those of Vostra-Vydrova et al. (2020) for White Shorthair goats and Brown Shorthair goat at 10.92 % and 10.07 % and 2 % and 5.3 %, respectively, and with those of Justinski et al. (2023) for 35 German sheep breeds, ranging from 3.5 % to 10 % and 0.8 % to 7.9 %, respectively.

Alleles that have experienced repeated inbreeding in the past are less likely to be harmful than those that have experienced inbreeding by descent (IBD) less frequently. This is because alleles persisting through purging are more likely to have neutral or beneficial effects on selected traits. Consequently, higher values of $F_{\mathrm{BAL}}$ , $F_{\mathrm{KAL}}$ , $F_{\mathrm{NEW}}$ , and $A_{\mathrm{HC}}$ are expected to influence phenotypic traits positively. Purging mitigates the adverse effects of inbreeding depression by reducing the impact of deleterious alleles, which typically manifest harmful effects when in a homozygous state. The approach entails reducing the presence of harmful alleles in ancestral populations through natural or artificial selection after inbreeding exposure. The degree of purging can be assessed by determining the linear regression slope between ancestral inbreeding coefficients and phenotypic characteristics. An optimistic estimate for ancestral inbreeding coefficients suggests evidence of purging. Ballou's concept of ancestral inbreeding provides a way to assess which individuals or populations are likely to carry fewer harmful genes. Higher values of this parameter suggest a greater likelihood of an individual having fewer detrimental genes. Based on this concept, the Marwari sheep breed population shows evidence of significant ancestral inbreeding ( $F_{\mathrm{BAL}}=4.09$ %) and is potentially less affected by inbreeding depression. In contrast, the mean estimates for $F_{\mathrm{KAL}}$ were much lower (0.28 %) because this focuses on homozygous alleles due to past common ancestry and only accounts for ancestral inbreeding through relationships. Unlike $F_{\mathrm{BAL}}$ , $F_{\mathrm{KAL}}$ remains zero for individuals with no classical inbreeding. The ancestral history coefficient ( $A_{\mathrm{HC}}$ ) estimates were higher (4.40 %) and closely aligned with $F_{\mathrm{BAL}}$ . The advantage of $A_{\mathrm{HC}}$ is that it provides a reliable measure of inbreeding in cases where selection against harmful recessive alleles is not entirely effective. The low level of $F_{\mathrm{NEW}}$ suggests that the selection and mating strategy of the breed result in similar changes in inbreeding despite the different sizes of the breeding populations across the individuals. Our results of ancestral inbreeding were comparable with the findings of Tohidi et al. (2023) for Iranian Holstein cattle, those of Justinski et al. (2023) for 35 German sheep breeds, those of Nguyen et al. (2023) for Mangalica pig breeds, and those of Posta et al. (2024) for the Danubia Alba rabbit.

**Figure 4:**
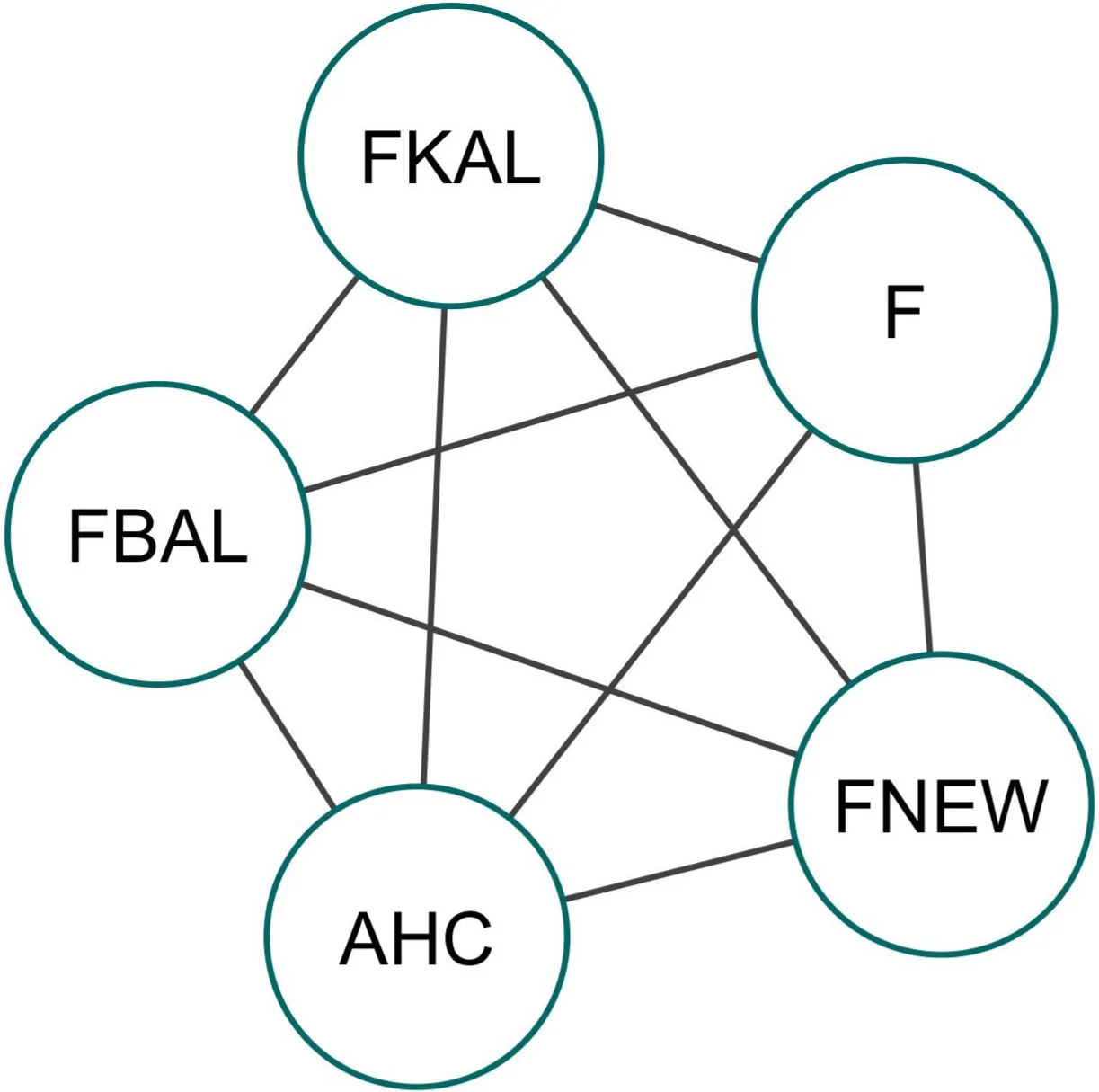
Network of significant associations obtained from the PCIT for different inbreeding estimates in Marwari lambs. Different inbreeding coefficients: F – pedigree-based inbreeding coefficient from all of the genealogy; $F_{\mathrm{BAL}}$ – pedigree-based inbreeding coefficient (Ballou, 1997); $F_{\mathrm{KAL}}$ – pedigree-based inbreeding coefficient (Kalinowski et al., 2000); $F_{\mathrm{NEW}}$ – recent pedigree-based inbreeding coefficient calculated from gene drop (Doekes et al., 2019); $A_{\mathrm{HC}}$ – ancestral history coefficient.

The correlation coefficient between F and $F_{\mathrm{NEW}}$ was nearly 1, suggesting that $F_{\mathrm{NEW}}$ constitutes a significant portion of F . This indicates that most classical inbreeding coefficients are made up of homozygous alleles that are identical by descent (IBD) for the first time within the pedigree. These findings contrast with McParland et al. (2009) but align with studies by Hinrichs et al. (2015) and Wirth et al. (2021). Differences in traits, environmental conditions, and pedigree depth may contribute to varying results. The PCIT network analysis helped clarify the relationships between the tested inbreeding coefficients, identifying 10 significant edges (Fig. 4). Significant positive correlations were observed among all inbreeding coefficients depicted in their inter-relationship. Classical and ancestral inbreeding were interconnected, as noted by Hinrichs et al. (2015). Factors like population structure and introgression made a difference in the correlation between inbreeding coefficients across studies and played key roles in this variability (Schäler et al., 2020).

**Figure 5:**
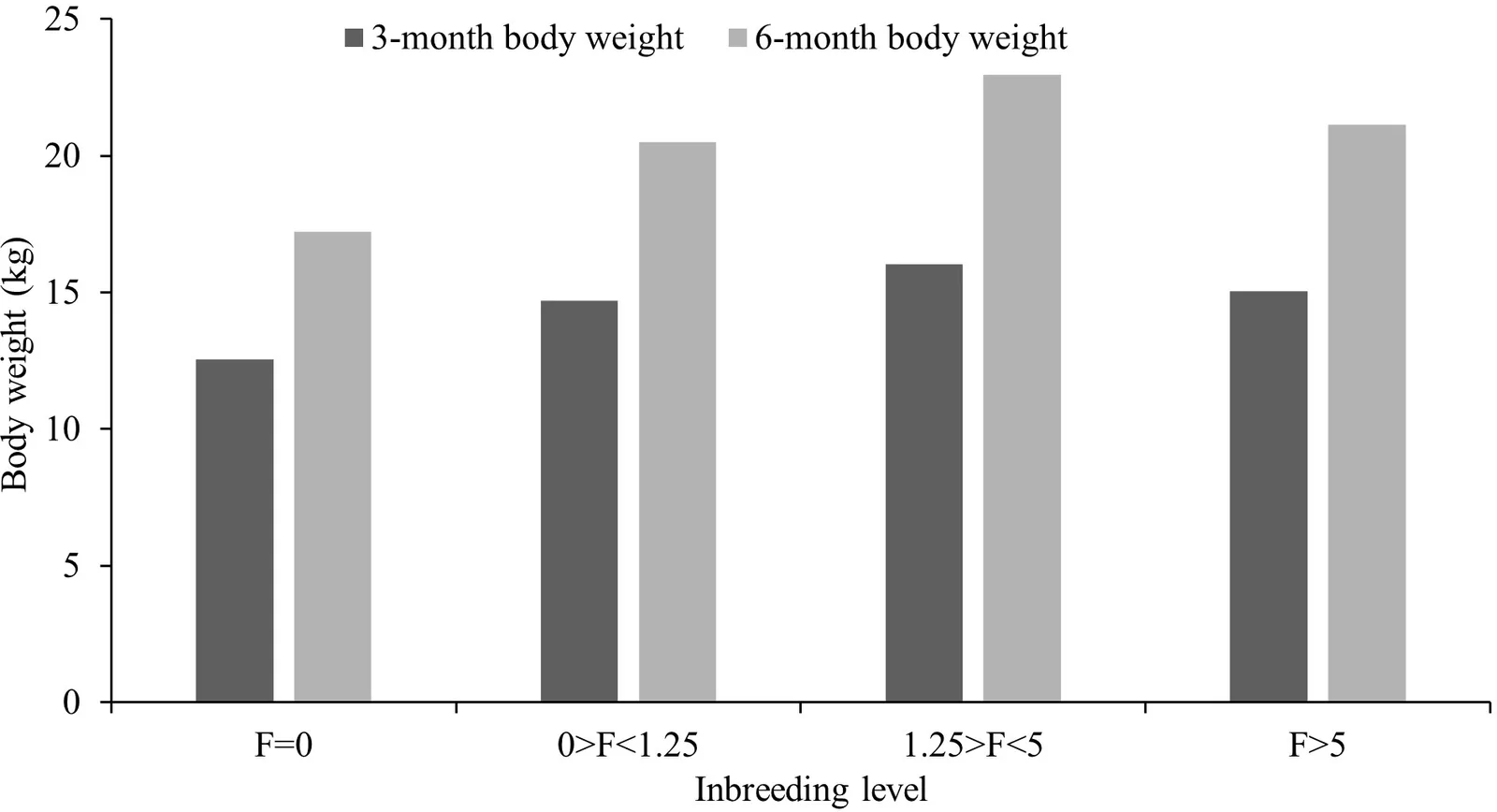
Grouping of Marwari lambs according to level of inbreeding coefficient for weaning and 6-month weight.

The observed level of inbreeding was within the usual range, indicating that appropriate breeding strategies were implemented effectively to minimize close breeding. Despite these attempts, inbreeding could not be avoided entirely due to other remote association routes in a closed flock. This may be the cause of the recent rise in inbreeding. The inbreeding coefficient ( F ) and the proportion of inbred animals increased linearly over time (Table 5). Similar low levels of inbreeding in different sheep breeds were reported by Negussie et al. (2002) for Horro sheep (0.78), Alsheikh (2005) for Egyptian Barki sheep (0.72), Prince et al. (2008) for Avikalin sheep (0.69), Kumar et al. (2008) for Chokla sheep (0.93), Arora et al. (2009) for Malpura sheep (0.66), Gowane et al. (2010) for Bharat Merino sheep (2.62), Cehyan et al. (2011) for Sakiz sheep (2.25), and Venkataramanan et al. (2016) for Nilagiri and Sandyno breeds of sheep (2.17 and 0.83, respectively). However, higher mean inbreeding was reported by MacKinnon (2003) for a crossbred sheep flock (3.8), having 50 % Dorset, 25 % Rambouillet, and 25 % Finn sheep, and by Nabi et al. (2021) for Corriedale sheep (18.1). Mandal et al. (2005) observed a similar increasing trend in Muzaffarnagari sheep, and Rzewuska et al. (2005) noted this trend in the Booroola flock. Over the periods, the number of inbred animals showed an overall increase, reflecting a greater incorporation of genes from common ancestry into a larger portion of the animal genome due to the closed nature of the flock. However, the proportion of inbred animals decreased after 2009 due to the introduction of outside rams. This proportion rose again until 2013 before experiencing a sharp decline in 2017, coinciding with another introduction of outside rams. These fluctuations indicate that including unrelated animals reduced inbreeding levels between 2010–2011 and 2016–2017. The results indicated that, despite many animals being inbred, most had very low inbreeding coefficients due to the making of sire lines and the restriction of males and females which are of the same sire line. The acquisition of outside animals reduced the proportion of inbred animals; however, it reached 82.38 % in the last period (2017–2020), suggesting an increase in the next generation. These results aligned with those of Arora et al. (2009), Gowane et al. (2010), and Venkataramanan et al. (2016) for their respective sheep breeds. The majority of animals were in the third class ( 1.25>F<5 ), while the fewest animals were in the fourth class ( F>5 ) for all traits examined. An increase in inbreeding coefficients ( F>5 ) negatively affected all traits studied, whereas a low level of inbreeding had a positive impact on most of the traits (Table 5). The inbreeding effect was particularly pronounced in small populations and was linked to reduced growth, decreased fitness, and increased incidence of genetic abnormalities (Windig et al., 2019). Numerous studies have also documented the adverse effects of inbreeding on various growth and fitness traits (Van Wyk et al., 2009; Chaudhari et al., 2023). The linear regression indicated that an increase of 1 % in inbreeding coefficient was associated with a change of 6, 79, 121, 146, and 116 g; 0.78, 0.38, and - 0.053 g d^−1^; 0.037, 0.008, and - 0.015; 1.427, 0.001, and - 0.277; and 0.003, 0.001, and - 0.001 in BWT, 3 MWT, 6 MWT, 9 MWT, and 12 MWT; ADG1, ADG2, and ADG3; KR1, KR2, and KR3; GE1, GE2, and GE3; and RGR1, RGR2, and RGR3, respectively. Among the significant effects observed, the first level with zero inbreeding showed a notable difference compared to the other classes, with inbreeding levels of 0 %–1.25 %, 1.25 %–5 %, and greater than 5 %. Controlled mating practices, focusing on sire lines and careful management, significantly influence all growth traits. The flock was genetically closed to preserve the purity of the indigenous breed, with limited introduction of outside germ plasm. Additionally, the population is continuously selected based on the growth performance of lambs at 6 months of age. Inbreeding had a highly significant and positive impact on all growth traits of the lambs (Fig. 5), except for KR3, GE3, and RGR3, where it showed a highly significant negative effect. In contrast, non-significant results were observed in ADG3, KR2, GE2, and RGR2. This depicted that feed conversion in the later stage of growth was affected by inbreeding and harmed the overall growth of Marwari sheep. The regression coefficient estimates for birth weight (0.002 kg %) are comparable with the reported estimates in the literature for various sheep breeds (Van Wyk et al., 1993; Boujenane and Chami, 1997; Analla et al., 1998; Mandal et al., 2005; Rzewuska et al., 2005).

**Table 5:** Grouping of Marwari lambs according to level of inbreeding coefficient for growth traits.

Trait	Level of inbreeding			
	F=0	0>F<1.25	1.25>F<5	F>5
BWT (kg)	2.87 ± 0.01 (3649)	3.06 ± 0.01 (2647)	3.14 ± 0.01 (4244)	3.04 ± 0.02 (571)
3 MWT (kg)	12.56 ± 0.06 (3153)	14.70 ± 0.07 (2537)	16.03 ± 0.05 (4052)	15.03 ± 0.15 (537)
6 MWT (kg)	17.23 ± 0.08 (2744)	20.51 ± 0.10 (2232)	22.96 ± 0.08 (3481)	21.12 ± 0.23 (465)
9 MWT (kg)	19.93 ± 0.09 (2384)	24.12 ± 0.12 (1819)	26.84 ± 0.10 (2560)	24.61 ± 0.29 (369)
12 MWT (kg)	22.06 ± 0.11 (2044)	26.70 ± 0.13 (1541)	29.66 ± 0.12 (2037)	26.82 ± 0.35 (306)
ADG1 (g d^−1^)	108.11 ± 0.59 (3125)	129.45 ± 0.69 (2532)	143.11 ± 0.52 (4045)	133.04 ± 1.57 (537)
ADG2 (g d^−1^)	53.70 ± 0.53 (2615)	64.04 ± 0.61 (2199)	76.54 ± 0.53 (3424)	67.83 ± 1.51 (452)
ADG3 (g d^−1^)	32.20 ± 0.31 (1826)	35.85 ± 0.40 (1492)	37.93 ± 0.37 (1967)	34.66 ± 0.92 (293)
KR1	15.90 ± 0.04 (3125)	17.02 ± 0.04 (2532)	17.70 ± 0.03 (4045)	17.20 ± 0.08 (537)
KR2	6.18 ± 0.05 (2615)	6.50 ± 0.05 (2199)	7.15 ± 0.04 (3424)	6.67 ± 0.11 (452)
KR3	3.10 ± 0.03 (1826)	3.04 ± 0.03 (1492)	2.95 ± 0.02 (1966)	2.90 ± 0.06 (293)
GE1	341.55 ± 1.87 (3125)	386.40 ± 2.22 (2532)	416.53 ± 1.76 (4045)	396.36 ± 4.77 (537)
GE2	39.88 ± 0.42 (2615)	39.95 ± 0.39 (2199)	43.73 ± 0.32 (3424)	41.17 ± 0.95 (452)
GE3	35.81 ± 0.38 (1826)	33.42 ± 0.40 (1492)	30.79 ± 0.32 (1966)	31.28 ± 0.85 (293)
RGR1	1.62 ± 0.004 (3125)	1.73 ± 0.005 (2532)	1.80 ± 0.003 (4045)	1.75 ± 0.01 (537)
RGR2	0.36 ± 0.003 (2615)	0.36 ± 0.003 (2199)	0.39 ± 0.002 (3424)	0.37 ± 0.007 (452)
RGR3	0.17 ± 0.002 (1826)	0.16 ± 0.001 (1492)	0.15 ± 0.001 (1966)	0.15 ± 0.004 (293)

BWT denotes birth weight, 3 MWT denotes weaning or 3-month weight, 6 MWT denotes 6-month weight, 9 MWT denotes 9-month weight, 12 MWT denotes 12-month weight, ADG1 denotes average daily gain from birth to 3 months, ADG2 denotes average daily gain from 3 to 6 months, ADG3 denotes average daily gain from 6 to 12 months, GE1 denotes growth efficiency from birth to 3 months, GE2 denotes growth efficiency from 3 to 6 months, GE3 denotes growth efficiency from 6 to 12 months, RGR1 denotes relative growth rate from birth to 3 months, RGR2 denotes relative growth rate from 3 to 6 months, and RGR3 denotes relative growth rate from 6 to 12 months. The number of observations is given in parentheses.

**Figure 6:**
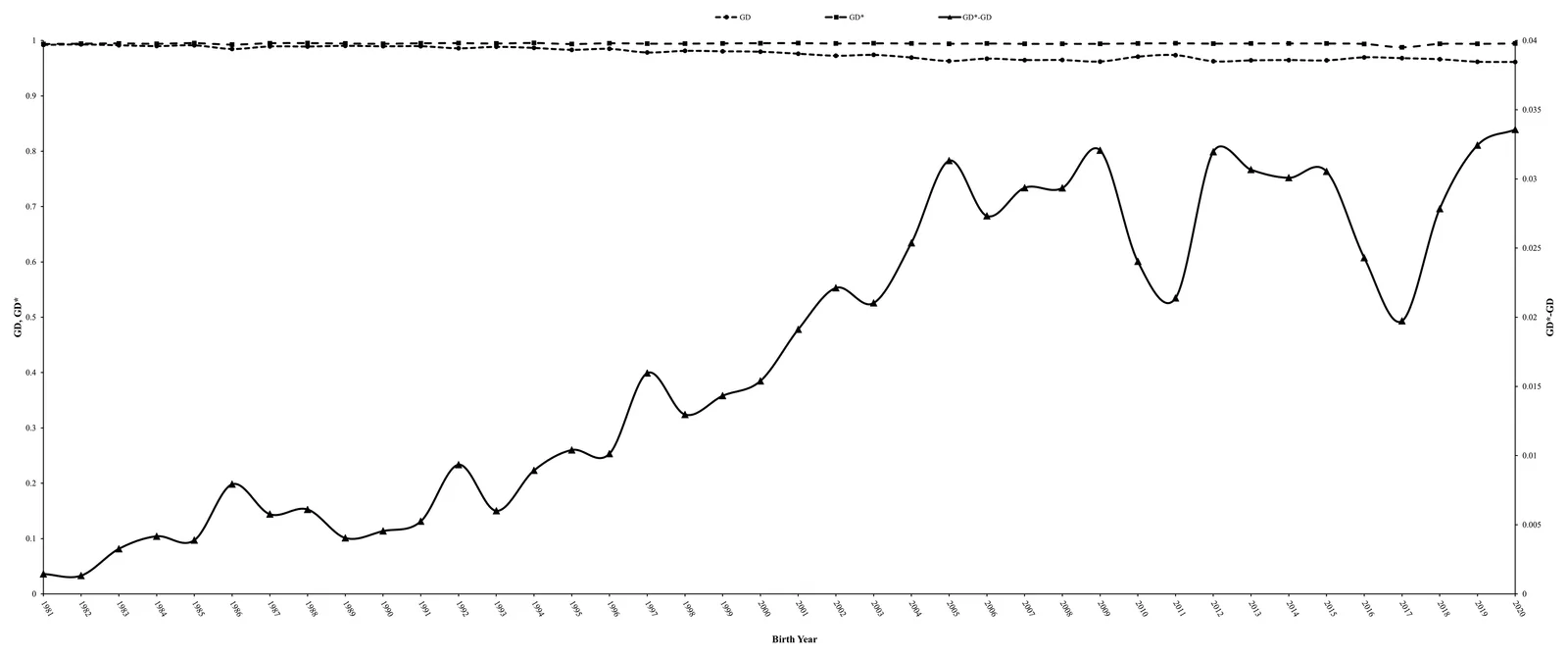
Evolution of genetic diversity in the reference population (GD) and in base population (GD^*^) and their difference (GD^*^ - GD) in the Marwari flock.

The impact of lamb inbreeding on weaning weight (0.015 kg %) aligns with the findings by Mandal et al. (2005) for Muzaffarnagari sheep, although a lower estimate was reported by Boujenane and Chami (1997). Nevertheless, Rashidi et al. (2014) and Patiabadi et al. (2017) found, for Markhoz goats and Iranian sheep, respectively, no significant negative impact on growth traits due to inbreeding. As the level of inbreeding increased, there was an increase in all studied growth traits except KR3, GE3, and RGR3, which exhibited an undesirable decrease. Significant adverse effects on body weight at 6, 9, and 12 months were also observed by Dorostkar et al. (2012) for Iranian Moghani sheep, by Gowane et al. (2014) for Malpura sheep, by Naghavian et al. (2016) for Shirva Kordi sheep, and by Venkataramanan et al. (2016) for Nilagiri sheep. The significant detrimental effect of inbreeding on body weight traits was observed by Mandal et al. (2004) for Muzaffarnagari sheep, Ceyhan et al. (2011) for Sakiz sheep, and Nabi et al. (2021) for Corriedale sheep. A significant detrimental effect of inbreeding on birth weight was observed by Gowane et al. (2010) for Bharat Merino sheep. Venkataramanan et al. (2016) found significant deleterious effect of individual inbreeding on 3 and 12 MWT and pre-ADG in Nilagiri sheep and on 12 MWT and post-ADG in Sandyno sheep. Non-significant effects of inbreeding on BWT and 3 and 6 MWT were observed by Kumar et al. (2008) for Chokla sheep and Arora et al. (2009) for Malpura sheep. It is crucial to prioritize conserving genetic diversity within the population through a national breeding policy. The genetic diversity within a population is necessary for adaptive capability and minimizes inbreeding depression for a long time. The ratio of $f_{g}/f_{a}$ explained that 36 % of the original ancestral genetic diversity was present in the reference population. Based on the estimated value of GD in the reference population relative to the base population, 3 % of the genetic diversity was lost over the studied period due to a reduction in heterozygosity over the years. The overall 2.3 % loss in genetic diversity (GD^*^ - GD) because of genetic drift accumulated across subsequent generations that are not founders in the Marwari sheep population (Fig. 6). This could be due to the closed nature of the flock and the restricted influx of outside animals. Previous reports on Muzaffarnagri sheep, Magra sheep, and Adani goat reported losses of 3.2 %, 2.2 %, and 3 %, respectively, in genetic variability within the base population (Mandal et al., 2020; Saran et al., 2024; Baneh et al., 2020).

## 5 Conclusions

The findings of this study revealed a loss of genetic diversity within the population; however, the level and trend of inbreeding in the closed flock of Marwari sheep remained within the recommended parameters. Over the years, a consistent pattern of inbreeding accumulation was observed, with a significant rise in the number of inbred animals due to structured scientific mating plans. Low inbreeding (1.25 % to 5 %) was beneficial and desirable for body weight traits. Inbreeding had no profound impact on growth characteristics, indicating that the institute's organizational structures and careful mating plans are on the right path. The steady increase in inbreeding over the years alerts us that subsequent mating should be more carefully prepared to prevent the mating of close relatives by introducing unrelated animals into the flock to maintain genetic variability. In addition, greater emphasis should also be placed on enhancing feed utilization efficiency traits to support the long-term sustainability and effectiveness of the breeding programme. Therefore, we propose optimizing scientific breeding practices to improve the genetic makeup, maintain genetic variability, keep inbreeding levels low, and prioritize efficiency-related traits in sheep production.

## Data Availability

The data that support the findings of this study are available from the corresponding author upon reasonable request.
